# The knowledge, training, and willingness of first year students in Xuzhou, China to perform bystander cardiopulmonary resuscitation: a cross-sectional study

**DOI:** 10.3389/fpubh.2024.1444970

**Published:** 2024-09-24

**Authors:** Zhaohui Qin, Shuyao Zheng, Chenxu Liu, Yuxin Ren, Ran Wang, Sitian Zhang, Xiao Gu, Yichen Li, Xianliang Yan, Tie Xu

**Affiliations:** ^1^Research Center for Medical and Health Emergency Rescue, The Second Clinical Medical School, Xuzhou Medical University, Xuzhou, China; ^2^The Second Clinical Medical School, Xuzhou Medical University, Xuzhou, China; ^3^School of Stomatology, Xuzhou Medical University, Xuzhou, China; ^4^The First Clinical Medical School, Xuzhou Medical University, Xuzhou, China

**Keywords:** cardiopulmonary resuscitation, knowledge, training, willingness, first year students

## Abstract

**Background:**

Bystander Cardiopulmonary Resuscitation (CPR) can significantly improve the rate of return of spontaneous circulation in patients with cardiac arrest. Since first year students with no specific academic background are energetic and quick to learn, many Chinese schools offer first-aid training course including CPR to them before they start school. However, data on CPR knowledge, training, and willingness among first year students are lacking in most regions of China, which makes the effectiveness of CPR training unknown.

**Objectives:**

To evaluate first year students’ knowledge level, training experience and rescue willingness for CPR of first year students in Xuzhou, and to analyze the influencing factors of CPR knowledge level and rescue willingness of first year students in Xuzhou.

**Design:**

A cross-sectional study.

**Participations:**

A total of 9,887 first year students from three universities in Xuzhou city were selected by multi-stage random cluster sampling method.

**Methods:**

A self-designed five-part structured questionnaire was used to investigate the knowledge, training and willingness of CPR among first year students. Independent sample *t*-test, *χ^2^*-test and Logistic regression were used for data analysis.

**Results:**

The average score of CPR knowledge was 2.44 (±1.60), 99.13% of the respondents were willing to participate in CPR training, and 66.25% had received CPR training. Respondents with rural household registration, relatives who had suffered from serious diseases, relatives engaged in the medical profession, good self-rated quality of life, CPR training, and willing to CPR training had higher CPR knowledge levels. 76.77% of the respondents were willing to perform bystander CPR. Women, those who had received CPR training, and those who were willing to receive CPR training were more willing to help patients with sudden cardiac arrest. Lack of first aid knowledge and skills (82.61%) was the biggest obstacle hindering respondents from performing rescue.

**Conclusion:**

Most of the first year students of Xuzhou University in China have CPR training experience and have a strong willingness to train. Most are willing to perform bystander CPR, but have a low knowledge level. Colleges and universities can adopt diversified training methods, make plans for regular CPR retraining and other strategies to improve the quality and effect of CPR training for college students.

## 1 Introduction

Out-of-hospital cardiac arrest (OHCA) is a prevalent issue in global health (1). A meta-analysis, encompassing 67 studies from diverse countries and regions indicates that the global incidence of OHCA varies between roughly 20 to 140 cases per 100,000 individuals. Correspondingly, the survival rate spans about 2 to 11% (2). As per the “China Cardiovascular Health and Disease Report 2022,” an estimated 330 million individuals in China were affected by cerebrovascular disease (often abbreviated as CVD), highlighting its immense impact in that region. Furthermore, CVD remains the primary cause of mortality among both urban and rural residents in China (3). Owing to the substantial prevalence of CVD patients, the annual occurrence of cardiac arrest in China is considerably high (4). As stated in the “China Cardiac Arrest and Cardiopulmonary Resuscitation Report (2022 edition),” the overall incidence of cardiac arrest in China in 2022 was approximately 97.1 per 100,000 individuals. Research has demonstrated that bystander Cardiopulmonary Resuscitation (CPR) serves as a significant determinant in enhancing the survival rate of OHCA cardiac arrest patients, and the rate of return of spontaneous circulation was significantly higher among patients who received bystander CPR compared to those who did not (5–7). Despite the increasing participation rate of bystander CPR in China in recent years (from 10.41% in 2013 to 19.36% in 2017), attributed to collaborative efforts by the Chinese government, medical institutions, and relevant social groups (8), it remains comparatively low when compared to other countries or regions, such as the United States, the United Kingdom, Australia and so on (9–11). The performance and quality of CPR are influenced by the level of CPR knowledge and CPR training experience possessed by bystanders (12, 13). Compared to other occupational groups, such as workers, farmers, and freelancers, college students exhibit a higher level of knowledge and a greater inclination to engage in bystander CPR (14). Consequently, comprehending the present state of training experience, training willingness, CPR knowledge level and rescue willingness among college students in China is essential. By encouraging college students to disseminate CPR knowledge to their peers and community, we can effectively promote the widespread adoption of emergency rescue practices.

The majority of prior research has primarily concentrated on college students residing in major urban centers such as Beijing with a population of over 21 million (15), Chongqing with nearly 31 million (16), and Wuhan with approximately 11 million residents (17). However, there is a scarcity of data on college students in non-major cities like Xuzhou, which has a significant population, with over 9 million residents, including a substantial student population of more than 240,000 across 13 universities (18). This survey takes first year students as the research object because they do not a have professional background. This uniformity helps to minimize potential biases that might arise from pre-existing professional knowledge or skills, such as those possessed by students in health-related fields. For instance, students with a medical or paramedical background might have a higher baseline of CPR knowledge and a different perspective on performing out-of-hospital CPR, which could skew the results if not accounted for. By focusing on first year students, we aim to capture a more accurate representation of CPR knowledge and willingness among individuals at the very start of their higher education journey, providing a solid baseline for CPR training needs and effectiveness. This is also the innovation of our topic selection. Therefore, this study aims to conduct a cross-sectional investigation into the existing levels of CPR knowledge, training experiences, training willingness and rescue willingness among first year students in Xuzhou. Simultaneously, we will also endeavor to explore the influencing factors of first year students’ CPR knowledge level and rescue willingness, to explore the factors affecting the behavior intention of bystander CPR and to provide a basis for further improving the knowledge level of college students’ CPR, improving the participation rate of bystander CPR, and improving the quality of bystander CPR.

## 2 Methods

### 2.1 Study setting and data collection

Our study was conducted from September to November 2022 among first year students in Xuzhou, China. Xuzhou is home to 13 universities, which include a variety of educational institutions ranging from research-oriented universities to vocational colleges, each offering different levels of higher education.

**Figure 1:**
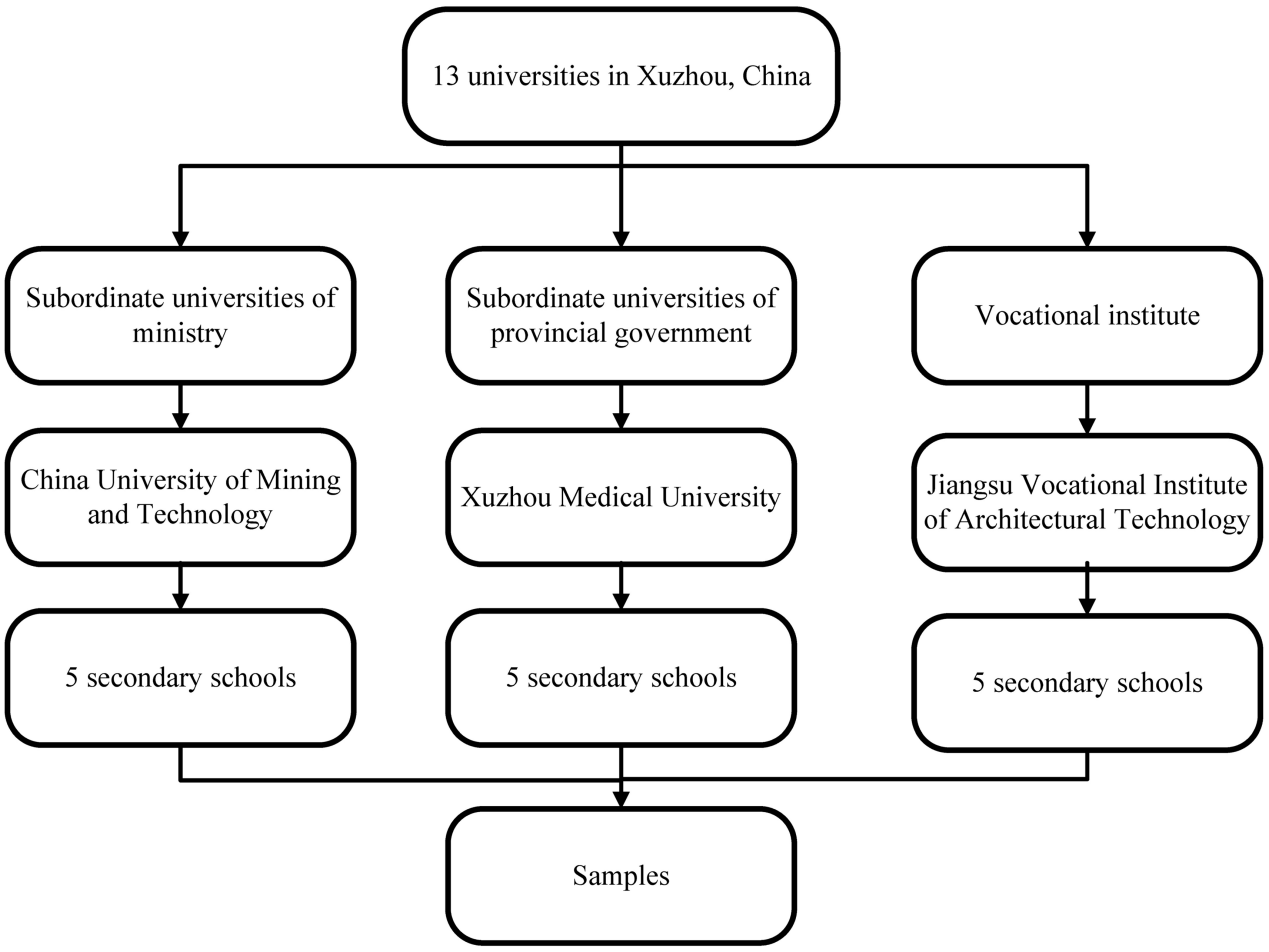
Sampling method.

This study employed a multi-stage random whole-group sampling method to ensure a diverse representation (Figure 1). In the initial stage, based on the different governing bodies, the 13 universities in Xuzhou were categorized into three groups: subordinate universities of ministry, subordinate universities of provincial government and vocational institute. To minimize potential biases or data interference that could arise from the unique characteristics of each university, such as their academic focus or student demographics, one university was randomly selected from each of the three categorized groups. In the subsequent stage, five secondary schools from each selected university were chosen randomly. First year students from these secondary schools underwent cluster sampling.

The CPR training referenced in this study adhered to the guidelines set forth by the American Heart Association (AHA), which is a widely recognized standard in CPR education. This curriculum, which includes the latest recommendations for chest compressions, rescue breaths, and the use of Automated External Defibrillators (AEDs), was consistently applied to all participants in the study to ensure uniformity in training quality and content. The use of a standardized curriculum also facilitated the comparison of participants’ knowledge and skills against established benchmarks.

Data collection was facilitated via the ‘Questionnaire Star’ platform. Participants accessed and completed the electronic questionnaire by scanning the QR (Quick Response) code. Importantly, all respondents were apprised of the purpose of the study’s objectives and provided their consent to partake in the study. For quality assurance, any questionnaire that was either incomplete or presented contradictory responses was deemed invalid.

### 2.2 Measures

A self-administered structured questionnaire was used for this survey, which was divided into five distinct sections, each designed to assess different aspects of the participants’ CPR knowledge and experiences (Table 1).

**Table 1:** Structure of the questionnaire sections.

Part	Questions
Characteristics of respondents	Gender
	Household registration
	Self-rated of the family financial situation
	History of major illness in relatives
	Relatives in the medical profession
CPR training status	CPR training experience
	When to participate in CPR training
	What organization conduct CPR training
	Forms of CPR training to attend
	Reasons for not having attended CPR training
CPR training willingness and requirements	Willingness to participate in CPR training
	What type of CPR training you would like to attend
	Desired frequency of CPR training
CPR knowledge	The role of automated external defibrillators
	Measures to take for respiratory and cardiac arrest
	The correct steps for CPR
	The ratio of CPR chest compressions to artificial respirations
	The correct hand position for chest compressions during CPR
	The depth of chest compressions during CPR
	The duration of a CPR cycle
CPR willingness	Willingness to perform bystander CPR
	Obstacles to perform bystander CPR

Section one focused on demographic information, establishing a baseline profile of the respondents. Section two evaluated the participants’ prior CPR training experiences, including the type and time of training received. Section three gauged the participants’ willingness to undergo CPR training and their preferences for training methods. Section four assessed the respondents’ knowledge of CPR through a series of factual questions. Finally, section five explored the participants’ attitudes toward performing bystander CPR and the barriers they perceived to providing such aid.

The questionnaire underwent a rigorous process of content validation by a panel of experts in the field of emergency medicine and public health, demonstrating a high level of reliability and validity (Cronbach’s *α* = 0.936, KMO = 0.967).

### 2.3 Data analysis

Data were analyzed using IBM SPSS 20.0 with the test level set at *α* = 0.05 and *P*<0.05 was considered a significant difference.

Continuous variables are described using means and standard deviations; counts and composition ratios represent categorical variables. The CPR knowledge section consisted of 7 questions, with 1 point for each correct answer and no points for incorrect answers, out of a total of 7 points. The respondents’ CPR knowledge score was calculated, and the results showed that the scores of the respondents presented a skewed distribution. Since the results showed poor CPR knowledge in the population as a whole, the level of CPR knowledge of the respondents was defined as the relative level of CPR knowledge in the population. Therefore, we classify a score > median as high and a score ≤ median as low. The *χ*^2^ test was employed to examine the disparities in categorical outcomes between respondents who received CPR training and those who did not. Logistic regression analysis investigated factors influencing individuals’ willingness to engage in CPR training.

## 3 Results

A comprehensive number of 9,976 questionnaires were completed for this survey. Following the elimination of 89 questionnaires due to their lack of responses or the presence of conflicting information, 9,887 questionnaires were deemed suitable for the final analysis.

### 3.1 Characteristics of the participants

The respondents had an average age of 18.20 ± 0.764, with a majority (over 99%) falling within the age range of 16 to 20 years old. Of the respondents, 60.99% (6030) identified as male. Additionally, 42.01% (4154) came from urban households, while 115 (1.16%) belonged to well-off families, 84.03% (8308) were from average family economic conditions, and 14.81% (1464) were classified as poor. Furthermore, 81.02% (8010) of the subjects reported having relatives with a history of major illness, and 36.77% (3635) of these relatives were employed in the medical profession (Table 2).

**Table 2:** Distribution of demographic characteristics of respondents’ CPR training experience.

	Total, *n*	Trained, *n* (%)	Untrained, *n* (%)	*χ^2^*	*p-*value
Gender				10.635	0.001
Male	6,030	3,920 (65.01)	2,110 (34.99)		
Female	3,857	2,630 (68.19)	1,227 (31.81)		
Household registration				62.202	<0.001
Urban	4,154	2,935 (70.65)	1,219 (29.35)		
Countryside	5,733	3,615 (63.06)	2,118 (36.94)		
Self-rated of the family financial situation				19.363	<0.001
Well-off	115	83 (72.17)	32 (27.83)		
Average	8,308	5,568 (67.02)	2,740 (32.98)		
Poor	1,464	899 (61.41)	565 (38.59)		
Self-rated quality of life				57.188	<0.001
Very Good	2045	1,463 (71.54)	582 (28.46)		
Good	3,745	2,532 (67.61)	1,213 (32.39)		
Normal	3,687	2,297 (62.45)	1,381 (37.55)		
Poor	316	198 (62.66)	118 (37.34)		
Very Poor	103	60 (58.25)	43 (41.75)		
History of major illness in relatives				10.407	0.001
No	1,877	5,366 (66.99)	2,644 (33.01)		
Yes	8,010	1,184 (63.08)	693 (36.92)		
Relatives in the medical profession				80.863	<0.001
No	6,252	3,938 (62.99)	2,314 (37.01)		
Yes	3,635	2,612 (71.86)	1,023 (28.14)		
Total	9,987	6,550 (66.25)	3,337 (33.75)		

### 3.2 CPR training experience

66.25% of the respondents (6550) received CPR training (Table 2). There were significant differences in CPR training rates between respondents of different genders, household registration, family economic condition, self-rated quality of life, history of major illness in relatives, and relatives in the medical profession (*p* ≤ 0.05) (Table 2).

Among respondents who had CPR training experience, 71.89% (4709) had attended CPR training 1 to 2 times, 21.33% (1397) had attended 3 to 4 times and 6.78% (444) had attended five times or more; 19.00% (1879) had participated in CPR training at primary school level, 43.76% (4327) at junior secondary school level, 52.23% (5164) at Senior high school level and 14.82% (1465) at undergraduate (or tertiary) level; 71.50% (4683) had attended CPR training in the form of face-to-face lectures, 57.31% (3754) had attended CPR training by watching online courses and 75.73% (4960) had attended CPR simulation exercises; schools (89.80%) were the main place where respondents attended CPR training. Respondents without experience of CPR training indicated that the biggest barrier to attending CPR training was not knowing how to attend (68.41%), followed by not having time to attend due to heavy academic commitments (27.81%), not seeing the need to attend (2.49%), and a small percentage (1.29%) not attending CPR training for other reasons.

### 3.3 CPR training willingness

Most respondents (99.13%) expressed willingness to participate in CPR training, while a negligible proportion (0.87%) indicated their lack of interest. Among those who were willing to attend CPR training, the majority (40.92% or 4,011 individuals) expected to receive training through regular simulation exercises, followed by face-to-face lectures (70.48%), online courses (51.33%), reading books and newspapers (36.40%), and accessing internet information (32.79%). Furthermore, it was found that 40.92% (4011) of the participants who were willing to attend CPR training anticipated receiving such training every month. Additionally, 45.01% (4111) of the respondents expected to have access to CPR training once per semester, while 14.48% (1419) believed that receiving CPR training once per school year would be satisfactory.

### 3.4 CPR knowledge

The proportion of correct answers on CPR knowledge is presented in Table 3. The mean score for CPR knowledge was 2.44 (±1.60), with a median score of 2, and the interquartile range was 3. The average score rate of CPR knowledge was 34.86% (2.44/7). Only 0.17% of respondents answered all questions correctly. Respondents with previous CPR training had a mean score of 2.717 (±1.505), significantly higher than those without CPR training (mean score = 1.899, *t* = −24.835, *p* < 0.001).

**Table 3:** Proportion of correct answers on CPR knowledge.

CPR knowledge	Total (*n*)	Percentage
The role of an AED	3,579	36.17
Measures to take in respiratory and cardiac arrest	5,487	55.50
The correct steps for CPR	2,736	27.67
The ratio of chest compressions to artificial respirations	2,601	26.31
The correct hand position for chest compressions during CPR	3,550	35.91
The depth of chest compressions during CPR	4,238	42.86
The duration of a CPR cycle	3,456	35.95

A total of 46.86% (*n* = 4,633) of the participants demonstrated a high level of CPR knowledge (CPR knowledge score > 2 points), while 53.14% (*n* = 5,234) exhibited a low level of CPR knowledge (CPR knowledge score ≤ 2 points). Logistic regression analysis revealed that individuals with family members who had experienced a major illness, those with relatives employed in the medical profession, those who had better self-rated quality of life are better, those who had received CPR training, and those who expressed a willingness to undergo CPR training displayed a higher level of CPR knowledge (Table 4).

**Table 4:** Logistic regression analysis: influence of demographic characteristics, CPR training experience and willingness on CPR knowledge level.

Characteristics	High level, *n* (%)	OR value (95%CI)	*P*-value
**Gender**			
Male	2,769 (45.92)	1.0	
Female	1,864 (48.33)	1.059 (0.975 to 1.151)	0.176
**Household registration**			
Countryside	2,566 (44.76)	1.0	
Urban	2,067 (49.76)	1.080 (0.991 to 1.178)	0.079
**History of major illness in relatives**			
No	3,703 (46.23)	1.0	
Yes	930 (49.55)	1.217 (1.096 to 1.352)	<0.001
**Relatives in the medical profession**			
No	2,750 (44.99)	1.0	
Yes	1,883 (51.80)	1.234 (1.133 to 1.345)	<0.001
**Self-rated of the family financial situation**			
Poor	647 (44.19)	1.0	
Average	3,921 (47.20)	0.930 (0.818 to 1.058)	0.464
Well-off	65 (56.52)	1.164 (0.777 to 1.746)	0.269
**Self-rated quality of life**			
Very poor	33 (32.04)	1.0	
Poor	116 (36.71)	1.166 (0.718 to 1.894)	0.536
Normal	1,642 (44.64)	1.682 (1.088 to 2.601)	0.019
Good	1,790 (47.80)	1.805 (1.163 to 2.803)	0.009
Very Good	1,052 (51.44)	2.001 (1.283 to 3.121)	0.002
**CPR training experience**			
No	1,094 (32.78)	1.0	
Yes	3,539 (54.03)	2.347 (2.149 to 2.562)	<0.001
**CPR training willingness**			
No	24 (27.91)	1.0	
Yes	4,609 (47.03)	2.163 (1.331 to 3.517)	<0.001

### 3.5 Willingness to perform bystander CPR

A significant majority of respondents, 76.77% (7590), expressed their willingness to perform bystander CPR on a patient experiencing sudden cardiac arrest, provided they possessed adequate mastery of CPR techniques. Conversely, a notable proportion of 23.23% exhibited hesitancy or diminished willingness to extend such assistance. Logistic regression analyses revealed that respondents who identified as female, possessed prior CPR training, had a high level of CPR knowledge, and were willing to undergo further CPR training displayed a higher inclination toward performing bystander CPR on a patient in sudden cardiac arrest (Table 5).

**Table 5:** Logistic regression analysis: influence of demographic characteristics, CPR knowledge level, CPR training experience and willingness on Willingness to perform bystander CPR.

Characteristics	Willing to rescue, *n* (%)	OR value (95%CI)	*P*-value
**Gender**			
Male	4,575 (75.87)	1.0	
Female	3,015 (78.17)	1.120 (1.016 to 1.235)	0.022
**Household registration**			
Urban	3,181 (76.58)	1.0	
Countryside	4,409 (76.91)	1.091 (0.987 to 1.206)	0.088
**History of major illness in relatives**			
No	6,174 (77.08)	1.0	
Yes	1,416 (75.44)	0.941 (0.835 to 1.062)	0.326
**Relatives in the medical profession**			
No	4,761 (76.15)	1.0	
Yes	2,829 (77.83)	1.051 (0.951 to 1.163)	0.330
**Self-rated of the family financial situation**			
Poor	1,103 (75.34)	1.0	
Average	6,396 (76.99)	1.009 (0.871 to 1.168)	0.907
Well-off	91 (79.13)	1.032 (0.634 to 1.680)	0.900
**Self-rated quality of life**			
Very poor	80 (77.67)	1.0	
Poor	221 (69.94)	0.555 (0.323 to 0.956)	0.034
Normal	2,795 (76.99)	0.704 (0.428 to 1.158)	0.166
Good	2,833 (75.65)	0.678 (0.410 to 1.121)	0.130
Very good	1,661 (81.22)	0.941 (0.565 to 1.568)	0.816
**CPR training experience**			
No	2,472 (74.08)	1.0	
Yes	5,118 (78.14)	1.186 (1.072 to 1.311)	0.001
**CPR training willingness**			
No	4,761 (76.15)	1.0	
Yes	2,829 (77.83)	5.641 (3.599 to 8.841)	<0.001
CPR knowledge level		1.	
Low level	3,927 (74.74)	1.0	
High level	3,663 (79.06)	1.205 (1.094 to 1.328)	<0.001

The lack of first aid knowledge and skills was the biggest obstacle that hindered respondents from performing bystander CPR followed by fear of secondary injury, practice, and dispute. Only a minority of respondents said it is none of their business. Meanwhile, some respondents expressed that they were willing to actively rescue at any time without hesitation (Table 6).

**Table 6:** Obstacle that hindered respondents from performing bystander CPR.

Obstacles	Total (%)
Lack of CPR knowledge and skills	8,168 (82.61%)
Fear of secondary injury	7,293 (73.76%)
Fear of practice	6,228 (62.99%)
Fear of dispute	5,185 (52.44%)
None of my business	1,433 (14.49%)
No scruples	1,500 (15.17%)

## 4 Discussion

This study was to determine the China Xuzhou first year students’ CPR training experience, training will, knowledge level and the current situation of rescue will. The results showed that more than half of the respondents had received CPR training, and more than 70% of them had attended CPR training only once or twice. Most of them were willing to participate in CPR training, but the level of CPR knowledge was low, with an average score of only 2.44 (out of 7). In addition, most respondents said they would be willing to perform bystander CPR if they had CPR skills.

In this survey, it was found that 66.25% of the respondents had participated in CPR training, surpassing the rates observed in Chongqing (33.6%) (16), Wuhan (14.6%) (17), and Xinjiang (44.9%) (19), while being comparable to the United States (65%) (20). However, it fell short of the rates observed in the United Kingdom (90%) (21) and Norway (89%) (22). Participation in CPR training is a prerequisite for acquiring CPR knowledge and proficiency in CPR skills. Studies have confirmed that individuals who have received CPR training are more inclined to perform CPR (23). Consequently, enhancing the proportion of individuals who have undergone CPR training stands is crucial to improving bystander CPR rates. Due to the concerted efforts of the Chinese government and relevant social entities (23), schools have emerged as the primary hub for comprehensive CPR training across all educational stages in China, fostering a continuous drive toward widespread dissemination of CPR technology and scientific knowledge. While commendable progress has been achieved, a discernible disparity remains compared to Europe, America and other developed countries. The respondents who had not participated in CPR training in this survey said that they did not know how to participate in CPR training was the biggest obstacle. Activities such as CPR week can be carried out to strengthen publicity and education, so as to increase the proportion of the population participating in CPR training.

In terms of the willingness aspect of CPR training, over 99% of the participants expressed their willingness to engage in CPR training, surpassing the survey of CPR training willingness among Chinese adults in 2019 (73.4%) (24), as well as previous research conducted in other countries (25). These findings indicate that college students, known for their strong learning abilities, exhibit the highest level of willingness to acquire CPR knowledge. Therefore, prioritising CPR training for college students is crucial in enhancing the overall CPR knowledge and skills within society. In addition, most respondents preferred CPR training through passive learning methods, such as simulation exercises, face-to-face lectures, and online courses. A minority of respondents indicated a desire for CPR training through active learning approaches, such as reading books, newspapers, and magazines and accessing network data. Several studies have demonstrated that traditional face-to-face CPR training (26), online computer-based CPR training (27), and virtual reality (VR) technology for CPR training (28) each possess distinct advantages. Therefore, embracing diverse methods of CPR training is conducive to enhancing the effectiveness and efficiency of such training.

Compared with the high rate of CPR training participation and the rate of willingness to participate in CPR training, the level of CPR knowledge in this survey was low. Only less than half of the respondents had a relatively high level of CPR knowledge, and the average score of CPR knowledge was only 34.86% (2.44/7). Less than 1 % of the respondents could answer all the questions correctly, similar to the results of a survey of college students in Chongqing (1%) (16). The correct answer rate for CPR knowledge showed the lowest percentage of respondents who could accurately identify the compression ventilation rate, aligning with findings from a study conducted on the general population in Turkey (29). Even for the question “Measures to take for respiratory and cardiac arrest” with the highest correct rate, only slightly more than half of the respondents could answer correctly, indicating that the overall CPR knowledge level of the respondents in this survey was low. The influencing factors of CPR knowledge level included “History of major illness in relatives,” “Relatives in the medical profession,” “Self-rated quality of life,” “CPR training experience” and “CPR training willingness.” A study of older adult respondents who were being treated in emergency wards found that most patients understood all components of CPR (30). As the results of our analysis show, the level of CPR knowledge is higher in those whose relatives have serious diseases. Since patients with serious diseases are more likely to have sudden cardiac arrest, doctors and nurses usually pay more attention to the popular science and training of CPR knowledge for such patients and their relatives. At the same time, out of concern and care for the relatives of the serious illness, the family members usually pay more attention to understanding the relevant knowledge. CPR is a required skills for medical practitioners (31), and even the CPR knowledge level of junior medical students is higher than that of non-professionals. Therefore, it is common sense that respondents with relatives engaged in the medical profession have higher CPR knowledge level. Some studies have found that self-rated health negatively correlates with depression (32), and self-rated health is an effective measure of quality of life (33). Therefore, people with better self-rated health are more likely to perform well in mental health and thus achieve better quality of life. This may explain why our analysis showed that participants with better self-rated quality of life were likelier to have a higher level of CPR knowledge. To give help to others is associated with better mental health (34); therefore, the self-rated a better quality of life were more willing to give help to others are more likely to focus on and take the initiative to learn CPR-related knowledge and skills, and therefore have higher CPR knowledge level. Several studies have confirmed that people who have been trained in CPR, whether in the medical profession or laypersons, have a higher level of CPR knowledge than those who have not (35, 36). At the same time, some studies also pointed out that even medical practitioners’ CPR knowledge and skill levels will decline after a period of CPR training (37, 38). Lack of regular CPR retraining May be one of the reasons for the low CPR knowledge level of the respondents in this survey, so it is particularly important to maintain a certain frequency of CPR retraining. The survey revealed that over 40% of the respondents expressed an expectation for monthly CPR training, while a similar proportion indicated a desire for CPR training on a semesterly basis. The findings of a randomized trial study aimed at determining the optimal frequency for high-quality CPR skills training demonstrated that CPR training once per month significantly improved CPR performance (39).

Although there is a limited overall understanding of CPR, over 70% of respondents indicated their willingness to perform bystander CPR if they were more proficient in CPR techniques. This is consistent with findings from surveys conducted among healthcare professionals in China (73.9%) (40) and community populations in the U.S. (77.8%) (41). Our study showed that women, those with prior CPR training experience, individuals open to further CPR education, and those with a higher CPR knowledge were more inclined to perform bystander CPR. Such trends echo results from prior surveys among medical staff in China. The predominant barrier hindering respondents from engaging in bystander CPR was a first-aid knowledge and skills deficiency.

Additionally, a significant majority expressed concerns about causing secondary harm during rescue efforts and potential disputes arising as a result, which closely mirror those obtained from a survey conducted in Taiwan (42). Over 60% of respondents admitted lacking confidence and fear regarding practical application. Notably, another survey involving Chinese students highlighted that the main hindrance to performing CPR on family members was fear of practice, whereas, for strangers, it was primarily driven by apprehension regarding potential conflicts stemming from secondary injuries incurred during resuscitation attempts (17). Only a small minority chose to perform bystander CPR without consideration indicating most respondents would carefully consider whether or not to administer assistance based on the specific circumstances encountered when faced with cardiac arrest patients to avoid unnecessary disputes.

The concerns most respondents expressed highlight a critical area where public perception is influenced by external factors. While our study did not specifically measure the impact of media exposure on first year students’ knowledge and willingness to perform CPR, existing literature suggests that media platforms, including social media, television, and movies, play a significant role in disseminating health information and influencing attitudes, particularly among younger demographics (43, 44). Media often serves as a primary source of information for the public, and its portrayal of CPR can either educate or mislead. For example, television and movies sometimes depict dramatic and inaccurate CPR scenarios that May exaggerate the risks of performing CPR, thereby increasing the apprehension of causing harm. On the other hand, responsible and accurate media coverage can demystify the process, reduce misconceptions, and encourage a more informed public response to cardiac arrest situations.

Given the influence of media, it is vital for educational institutions and healthcare organizations to partner with media professionals to promote accurate CPR knowledge and techniques. This can be achieved through educational campaigns, public service announcements, and engaging content that addresses common misconceptions and emphasizes the importance of CPR in life-saving situations., Considering the high level of media consumption among young people, it would be valuable to assess the extent to which first-year students are influenced by media in their understanding and attitudes toward CPR and assess the impact of such content on their knowledge, attitudes, and willingness to perform CPR in future research.

### 4.1 Limitation

The limitation of this investigation is that, first of all, this study takes first year students in Xuzhou as the research object, which cannot reflect the situation of the whole first year students, nor can it represent the situation in other regions of China. In addition, this study is a cross-sectional study, and it is not certain whether CPR training affects on improving the CPR knowledge level of the respondents. Further studies are needed to confirm this. Finally, the self-designed questionnaire used in this survey May still not be perfect enough to reflect the participants’ situation fully.

## 5 Conclusion

Most of first year students in Xuzhou, China, with CPR training experience and strong training willingness are willing to implement the bystander CPR. However, most of them only received one or two CPR trainings; due to a lack of periodic CPR training, their knowledge level is relatively low. This also leads to the lack of first-aid knowledge and skills as the main obstacle to implementing bystander CPR. Colleges and universities should further promote the popularization of CPR knowledge and skills based on the achievements so far, adopt diversified training methods, and formulate plans for regular CPR retraining to improve the quality and effect of CPR training for college students. At the same time, based on the experience of college students’ CPR training for the whole population to improve the efficiency and effect of large-scale CPR training.

## Data Availability

The raw data supporting the conclusions of this article will be made available by the authors, without undue reservation.
